# New Insights Into the Threshold Values of Multi-Locus Sequence Analysis, Average Nucleotide Identity and Digital DNA–DNA Hybridization in Delineating *Streptomyces* Species

**DOI:** 10.3389/fmicb.2022.910277

**Published:** 2022-05-31

**Authors:** Siren Hu, Kaiqin Li, Yifei Zhang, Yinfeng Wang, Li Fu, Yan Xiao, Xinke Tang, Jian Gao

**Affiliations:** ^1^School of Life and Health Sciences, Hunan University of Science and Technology, Xiangtan, China; ^2^Key Laboratory of Ecological Remediation and Safe Utilization of Heavy Metal-Polluted Soils, College of Hunan Province, Xiangtan, China

**Keywords:** new insights, ANIm, dDDH, MLSA, *Streptomyces*

## Abstract

Multi-locus sequence analysis (MLSA) has been proved to be a useful method for *Streptomyces* identification and MLSA distance of 0.007 is considered as the boundary value. However, we found that MLSA distance of 0.007 might be insufficient to act as a threshold according to the correlations among average nucleotide identity based on MuMmer ultra-rapid aligning tool (ANIm), digital DNA–DNA hybridization (dDDH) and MLSA from the 80 pairs of *Streptomyces* species; in addition, a 70% dDDH value did not correspond to a 95∼96% ANIm value but approximately to 96.7% in the genus *Streptomyces*. Based on our analysis, it was proposed that when the MLSA distance value between a novel *Streptomyces* and a reference strain was < 0.008, the novel strain could be considered as a heterotypic synonym of the reference strain; when the MLSA distance value was ≥ 0.014, the novel strain could be regarded as a new *Streptomyces* species; when the MLSA distance value was between 0.008 and 0.014 (not included), the dDDH or ANIm value between a new strain and a reference strain must be calculated in order to determine the taxonomic status of a novel strain. In this context, a 70% dDDH or 96.7% ANIm value could act as the threshold value in delineating *Streptomyces* species, but if the dDDH or ANIm value was less than but close to 70 or 96.7% cut-off point, the taxonomic status of a novel strain could only be determined by a combination of phenotypic characteristics, chemotaxonomic characteristics and phylogenomic analysis.

## Introduction

In current prokaryote systematics, the classification of *Bacteria* and *Archaea* is based on a polyphasic taxonomic approach, comprised of phenotypic, chemotaxonomic and genotypic data, as well as phylogenetic information (Schleifer, 2009). Of these, the classical DNA–DNA hybridization (DDH) technology plays a key role in novel species identification. Although DDH has been the “gold standard” for bacterial species demarcation over the last 50 years, its procedures are known to be labor-intensive, error-prone and do not allow the generation of cumulative databases. Thus, there has been an urgent need for an alternative genotype-based standard (Stackebrandt et al., 2002; Gevers et al., 2005). With the rapid progress in the area of genome sequencing technology, many efforts have been made to develop a bioinformatic method to replace classical DDH for differentiating species. These efforts were mainly focused on devising values analogous to DDH values, such as genome BLAST distance phylogeny (GBDP) (Henz et al., 2005), average nucleotide identity (ANI) (Konstantinidis et al., 2006), maximal unique matches index (MUMi) (Deloger et al., 2009) and digital DNA–DNA hybridization (dDDH) (Auch et al., 2010). At present, ANI or dDDH has been most widely used as a gold standard for species delineation. Unfortunately, over the last two decades, even though a lot of efforts have been made to obtain genome data for prokaryotic organism, only approximately 2.1% of the global prokaryotic taxa are represented by sequenced genomes (Zhang et al., 2020). As far as *Streptomyces* species are concerned, genome data of about 30% type species with validly published and correct names are still unavailable at the time of writing this article^1^. In contrast, the nearly entire database of 16S rRNA gene sequences is available for the type strains of the genus *Streptomyces*. Nevertheless, when sequence similarity of 16S rRNA gene between two strains is over 97% (Stackebrandt et al., 2002; Tindall et al., 2010), it is hard to differentiate two species using 16S rRNA gene sequences alone. Therefore, in the modern classification of *Streptomyces*, 16S rRNA gene sequence similarity, ANI and dDDH values are usually used in combination to assess phylogenetic position of a novel species, and only species exhibiting ≥ 98.7% 16S rRNA gene sequence similarity are required to calculate ANI or dDDH values (Konstantinidis and Tiedje, 2005; Stackebrandt and Ebers, 2006; Meier-Kolthoff et al., 2013). If genome sequence data of the type strains with ≥ 98.7% 16S rRNA gene sequence similarity are unavailable, it is recommended to obtain their genome sequences, not only to measure ANI and dDDH but also to extend and improve the public genome database for taxonomic purposes (Chun et al., 2018). However, even though the whole-genome sequencing is accessible to most of the microbial taxonomists at the present, it is still time-consuming and costly. Thus, it is of great significance for many microbial taxonomists to find out an alternative to ANI or dDDH. In contrast to ANI or dDDH, multilocus sequence analysis (MLSA) based on housekeeping genes is a simple and low-cost approach and has been proved to be a useful method for identification of *Streptomyces* species (Guo et al., 2008; Rong et al., 2009, 2010; Rong and Huang, 2010, 2012; Labeda et al., 2017). In an early comparative study between DDH and MLSA, Rong and Huang proposed that the MLSA evolutionary distance of 0.007 could act as the threshold value in delineating *Streptomyces* species (Rong et al., 2010). However, our recent findings are somewhat different from their conclusion. In addition, we also found that the 70% dDDH value was not equivalent to the 95∼96% ANI value in the genus *Streptomyces*. In the present work, new insights into the threshold values of MLSA, ANI and dDDH in delineating *Streptomyces* species were provided based on the correlation among ANI, dDDH and MLSA from 80 pairs of *Streptomyces* species (including heterotypic synonyms).

## Materials and Methods

### Source of Genome Data and Type Strains

A total number of 95 genomes from type *Streptomyces* species with validly published names were downloaded from the GenBank database. The complete genome list is shown in Supplementary Table 1. All anomalous assemblies were discarded. The type strains *S*. *albidoflavus* CGMCC 4.1291^T^, *S*. *canarius* CGMCC 4.1581^T^, *S*. *castelarensis* CGMCC 4.3570^T^, *S*. *chartreusis* CGMCC 4.1639^T^, *S. corchorusii* CGMCC 4.1592^T^, *S*. *melanosporofaciens* CGMCC 4.1742^T^, *S*. *mirabilis* CGMCC 4.1988^T^, and *S*. *olivochromogenes* CGMCC 4.2000^T^ were purchased from China General Microbiological Culture Collection Centre (CGMCC), while *S. koyangensis* JCM 14915^T^ and *S. osmaniensis* JCM 17656^T^ were from Japan Collection of Microorganisms (JCM).

### Correlation Among ANIm, dDDH and MLSA

Given that ANIm (average nucleotide identity based on MuMmer ultra-rapid aligning tool) provides more credible results when the pair of genomes compared share a high degree of similarity (ANI > 90%) (Richter and Rosselló-Móra, 2009), the ANIm value rather than the ANIb (ANI based on the BLAST algorithm) value was selected for comparative analysis in the current work. The calculations of ANIm and dDDH values were performed by using the JSpeciesWS online service (Richter et al., 2015) and the Genome-to-Genome Distance Calculator (Meier-Kolthoff et al., 2013), respectively. For calculating dDDH value, Formula 2 was used. The sequences of five protein-coding genes (*atp*D, *gyr*B, *rec*A, *rpo*B, and *trp*B) were directly drawn from draft genome sequences. After trimmed manually using methods of Rong and Huang (2012), five gene sequences were concatenated head-to-tail in-frame in the order of *atp*D, *gyr*B, *rec*A, *rpo*B, and *trp*B. The MLSA evolutionary distances between a set of type strains were calculated according to Kimura’s two-parameter model (Kimura, 1980). The datasets for coherence analysis among ANIm, dDDH and MLSA were processed by Origin Pro 9.0. Coefficients of determination (R^2^) among ANIm, dDDH and MLSA were calculated by exponential regression analysis.

### Phenotypic Characterization

The cultural characteristics of ten tested strains, i.e., *S. albidoflavus* CGMCC 4.1291^T^, *S. canarius* CGMCC 4.1581^T^, *S. castelarensis* CGMCC 4.3570^T^, *S. chartreusis* CGMCC 4.1639^T^, *S. corchorusii* CGMCC 4.1592^T^, *S. koyangensis* JCM 14915^T^, *S. melanosporofaciens* CGMCC 4.1742^T^, *S. mirabilis* CGMCC 4.7010^T^, *S. olivochromogenes* CGMCC 4.2000^T^, and *S. osmaniensis* JCM 17656^T^, were evaluated on ISP serial agar media (Shirling and Gottlieb, 1966) following incubation at 28°C for 14 days. The colors of colonies and soluble pigments were determined according to the Color Standards and Color Nomenclature (Ridgway, 1912). A range of physiological and biochemical tests were carried out according to Li et al.’s methods Li et al. (2020). Tolerance to different temperatures (4, 10, 15, 20, 25, 28, 30, 37, 40, and 45°C) was tested on ISP2 agar for 14 days. Enzyme-activity tests were carried out using API-ZYM test system (France) according to the manufacturer’s instructions. Other physiological characteristics including starch hydrolysis, gelatin liquefaction, milk coagulation and peptization, melanin production, Tweens (20, 40, 60, and 80) degradation, H_2_S production and nitrate reduction were performed according to the methods described by Xu et al. (2007). All these experiments were carried out in triplicate, and all these strains were grown under the same conditions for parallel comparison.

### Chemotaxonomic Characterization

Cells were collected for chemotaxonomic analysis by centrifugation from five strains cultured at 28°C in TSB medium for 7 days on a rotary shaker and then washed twice with distilled water. The diaminopimelic acid (DAP) isomer and whole-cell sugar compositions were analyzed using TLC according to the procedures described by Lechevalier and Lechevalier (1970) and Hasegawa et al. (1983). Cellular fatty acids analysis was carried out by China Center of Industrial Culture Collection (CICC) according to the protocol of the Sherlock Microbial Identification system [MIDI system, version 6.0B, MIDI (2005)]. Menaquinones were extracted according to Collins et al. (1977) and analyzed by HPLC (Wu et al., 1989). The polar lipids were extracted and identified by the method of Kates (1986).

### Phylogenomic Analysis

The genome sequences of five pairs of *Streptomyces* and relevant reference strains for phylogenomic analysis were retrieved from NCBI database. Phylogenomic analysis was carried out using the Type (Strain) Genome Server (Meier-Kolthoff and Göker, 2019). A phylogenetic tree was inferred with FastME (Lefort et al., 2015) from the Genome BLAST Distance Phylogeny (GBDP) distances calculated from genome sequences.

## Results and Discussion

There is no doubt that MLSA plays an extremely important role in identifying *Streptomyces* species (Labeda et al., 2017). However, recently, during identifying a novel strain of endophytic *Streptomyces* from a medicinal plant, we found that the MLSA evolutionary distance between *S. stelliscabiei* DSM 41803^T^ and *S. bottropensis* ATCC 25435^T^ was 0.011 (greater than the 0.007 critical point proposed for delineating *Streptomyces* species), suggesting that they should belong to different genomic species (Rong and Huang, 2012). This result was contradictory to Madhaiyan et al.’s (2020) conclusion that *S. stelliscabiei* is a later heterotypic synonym of *S. bottropensis* based on comparative genomic analysis. Is this case an exceptional one? To answer this question, firstly, we calculated ANIm values among the majority of validly published *Streptomyces* species whose genomes are available. Then, all strain pairs, whose ANIm values are greater than or equal to 90%, were collected for subsequent analysis. Finally, MLSA, dDDH and ANIm values of a total of 80 pairs of *Streptomyces* species were randomly selected from the above strain pairs to compare with each other (Table 1). Results indicated that besides *S. stelliscabiei* DSM 41803^T^ and *S. bottropensis* ATCC 25435^T^, there were six strain pairs, i.e., *S. antimycoticus* NBRC 12839^T^ and *S. melanosporofaciens* DSM 40318^T^, *S. canarius* JCM 4733^T^ and *S. olivaceoviridis* JCM 4499^T^, *S. durhamensis* NRRL B-3309^T^ and *S. filipinensis* JCM 4369^T^, *S. glebosus* NBRC 13786^T^ and *S. platensis* DSM 40041^T^, *S. griseorubens* JCM 4383^T^ and *S. matensis* JCM 4277^T^, and *S. melanosporofaciens* DSM 40318^T^ and *S. sporoclivatus* NBRC 100767^T^, in which not only the MLSA evolutionary distance in each pair was higher than 0.007, but also the dDDH and ANIm values were more than the 70% or 95∼96% cut-off points recommended for delineating species (Stackebrandt and Goebel, 1994; Richter and Rosselló-Móra, 2009), respectively. In addition, there were four strain pairs, i.e., *S. canarius* JCM 4733^T^ and *S. corchorusii* DSM 40340^T^, *S. castelarensis* NRRL B-24289^T^ and *S. melanosporofaciens* DSM 40318^T^, *S. chartreusis* ATCC 14922^T^ and *S. osmaniensis* OU-63^T^, and *S. mirabilis* JCM 4551^T^ and *S. olivochromogenes* DSM 40451^T^, in which the MLSA evolutionary distances in each pair were greater than or equal to 0.007, and the dDDH values were lower than 70%, but the ANIm values were over 95∼96%. All these data indicated that the MLSA evolutionary distance of 0.007 might be insufficient to act as the threshold value in delineating *Streptomyces* species.

**TABLE 1:** ANIm, MLSA, and dDDH values among 78 pairs of type *Streptomyces* species including heterotypic synonyms.

No.	Species 1	Species 2	ANIm	MLSA	dDDH
1	*S. flavovariabilis* NRRL B-16367^T^	*S. variegatus* NRRL B-16380^T^	99.99	0.000	99.8
2	*S. almquistii* NRRL B-1685^T^	*S. albus* NRRL B-1811^T^	99.95	0.000	99.7
3	*S. phaeogriseichromatogenes* DSM 40710^T^	*S. griseofuscus* NRRL B-5429^T^	99.48	0.001	95.3
4	*S. asterosporus* DSM 41452^T^	*S. aureorectus* DSM 41692^T^	99.19	0.002	92.8
5	*S. aureorectus* DSM 41692^T^	*S. calvus* CECT 3271^T^	99.20	0.002	92.8
6	*S. asterosporus* DSM 41452^T^	*S. calvus* CECT 3271^T^	99.16	0.001	92.9
7	*S. plicatus* JCM 4504^T^	*S. vinaceusdrappus* JCM 4529^T^	99.23	0.002	93.6
8	*S. plicatus* JCM 4504^T^	*S. geysiriensis* JCM 4962^T^	98.97	0.001	91.2
9	*S. geysiriensis* JCM 4962^T^	*S. vinaceusdrappus* JCM 4529^T^	99.01	0.002	91.4
10	*S. hygroscopicus subsp. hygroscopicus* NBRC 13472^T^	*S. endus* NBRC 12859^T^	98.94	0.000	90.1
11	*S. puniceus* NRRL ISP-5083^T^	*S. floridae* NRRL 2423^T^	98.83	0.002	89.8
12	*S. sporoclivatus* NBRC 100767^T^	*S. antimycoticus* NBRC 12839^T^	98.75	0.003	88.6
13	*S. puniceus* NRRL ISP-5083^T^	*S. californicus* NRRL B-2098^T^	98.63	0.001	87.6
14	*S. californicus* NRRL B-2098^T^	*S. floridae* NRRL 2423^T^	98.62	0.002	87.5
15	*S. griseorubens* JCM 4383^T^	*S. matensis* JCM 4277^T^	97.95	0.008	80.9
16	*S. galilaeus* ATCC 14969^T^	*S. bobili* NRRL B-1338^T^	97.74	0.004	79.6
17	*S. glebosus* NBRC 13786^T^	*S. platensis* DSM 40041^T^	97.78	0.008	79.4
18	*S. castelarensis* NRRL B-24289^T^	*S. sporoclivatus* NBRC 100767^T^	97.49	0.006	76.2
19	*S. castelarensis* NRRL B-24289^T^	*S. antimycoticus* NBRC 12839^T^	97.47	0.005	75.8
20	*S. olivaceoviridis* JCM 4499^T^	*S. canarius* JCM 4733^T^	97.41	0.009	76.0
21	*S. griseofuscus* NRRL B-5429^T^	*S. murinus* NRRL B-2286^T^	97.15	0.004	74.5
22	*S. phaeogriseichromatogenes* DSM 40710^T^	*S. murinus* NRRL B-2286^T^	97.14	0.006	74.7
23	*S. costaricanus* DSM 41827^T^	*S. murinus* NRRL B-2286^T^	97.08	0.005	73.9
24	*S. filipinensis* JCM 4369^T^	*S. durhamensis* NRRL B-3309^T^	96.97	0.010	72.9
25	*S. melanosporofaciens* DSM 40318^T^	*S. sporoclivatus* NBRC 100767^T^	96.91	0.012	72.2
26	*S. antimycoticus* NBRC 12839^T^	*S. melanosporofaciens* DSM 40318^T^	96.90	0.010	72.0
27	*S. olivaceoviridis* JCM 4499^T^	*S. corchorusii* DSM 40340^T^	96.88	0.006	71.4
28	*S. recifensis* NRRL B-3811^T^	*S. griseoluteus* JCM 4765^T^	96.86	0.005	72.4
29	*S. stelliscabiei* DSM 41803^T^	*S. bottropensis* ATCC 25435^T^	96.86	0.011	70.9
30	*S. costaricanus* DSM 41827^T^	*S. griseofuscus* NRRL B-5429^T^	96.74	0.004	70.9
31	*S. costaricanus* DSM 41827^T^	*S. phaeogriseichromatogenes* DSM 40710^T^	96.73	0.003	70.9
32	*S. canarius* JCM 4733^T^	*S. corchorusii* DSM 40340^T^	96.69	0.007	69.5
33	*S. castelarensis* NRRL B-24289^T^	*S. melanosporofaciens* DSM 40318^T^	96.57	0.009	68.7
34	*S. chartreusis* ATCC 14922^T^	*S. osmaniensis* OU-63^T^	96.40	0.008	68.8
35	*S. mirabilis* JCM 4551^T^	*S. olivochromogenes* DSM 40451^T^	96.23	0.011	67.0
36	*S. albidoflavus* NRRL B-1271^T^	*S. koyangensis* VK-A60^T^	95.90	0.009	64.7
37	*S. longwoodensis* DSM 41677^T^	*S. lasalocidi* X-537^T^	95.47	0.008	61.8
38	*S. bauhiniae* Bv016^T^	*S. griseoluteus* JCM 4765^T^	95.27	0.014	60.6
39	*S. rhizosphaericola* 1AS2c^T^	*S. cavourensis* DSM 41795^T^	95.21	0.012	59.8
40	*S. recifensis* NRRL B-3811^T^	*S. bauhiniae* Bv016^T^	95.21	0.015	60.7
41	*S. xiaopingdaonensis* L180^T^	*S. sulphureus* DSM 40104^T^	95.20	0.019	59.7
42	*S. bauhiniae* Bv016^T^	*S. seoulensis* KCTC 9819^T^	95.18	0.013	60.2
43	*S. aquilus* GGCR-6^T^	*S. antibioticus* DSM 40234^T^	94.86	0.017	58.4
44	*S. achromogenes subsp. achromogenes* NRRL B-2120^T^	*S. achromogenes* subsp. *rubradiris* JCM 4955^T^	94.75	0.016	56.2
45	*S. parvus* NRRL B-1455^T^	*S. mediolani* NRRL WC-3934^T^	94.53	0.029	56.1
46	*S. recifensis* NRRL B-3811^T^	*S. seoulensis* KCTC 9819^T^	94.47	0.019	56.6
47	*S. galbus* JCM 4639^T^	*S. lasalocidi* X-537^T^	94.44	0.009	55.4
48	*S. seoulensis* KCTC 9819^T^	*S. griseoluteus* JCM 4765^T^	94.39	0.019	55.8
49	*S. longwoodensis* DSM 41677^T^	*S. galbus* JCM 4639^T^	94.33	0.010	55.0
50	*S. ochraceiscleroticus* NRRL ISP-5594^T^	*S. violens* NRRL ISP-5597^T^	94.24	0.014	54.5
51	*S. reniochalinae* LHW50302^T^	*S. diacarni* LHW51701^T^	93.51	0.018	50.2
52	*S. phaeoluteigriseus* DSM 41896^T^	*S. bobili* NRRL B-1338^T^	93.47	0.021	50.7
53	*S. violaceusniger* NBRC 13459^T^	*S. antioxidans* MUSC 164^T^	93.34	0.027	48.1
54	*S. sedi* JCM 16909^T^	*S. zhaozhouensis* CGMCC 4.7095^T^	93.21	0.021	48.7
55	*S. qaidamensis* S10^T^	*S. variegatus* NRRL B-16380^T^	93.10	0.037	49.0
56	*S. tirandamycinicus* HNM0039^T^	*S. spongiicola* HNM0071^T^	92.95	0.020	45.1
57	*S. flavovariabilis* NRRL B-16367^T^	*S. iakyrus* NRRL ISP-5482^T^	92.86	0.033	47.9
58	*S. violaceorubidus* NRRL B-16381^T^	*S. rubrogriseus* NBRC 15455^T^	92.80	0.019	47.3
59	*S. hawaiiensis* ATCC 12236^T^	*S. tuirus* JCM 4255^T^	92.75	0.031	47.6
60	*S. platensis* DSM 40041^T^	*S. libani subsp. libani* NBRC 13452^T^	92.58	0.033	45.9
61	*S. glebosus* NBRC 13786^T^	*S. libani subsp. libani* NBRC 13452^T^	92.55	0.033	45.9
62	*S. coelicoflavus* NBRC 15399^T^	*S. rubrogriseus* NBRC 15455^T^	92.48	0.018	45.7
63	*S. diastaticus subsp. ardesiacus* NBRC 15402^T^	*S. coelicoflavus* NBRC 15399^T^	92.34	0.023	45.4
64	*S. libani subsp. libani* NBRC 13452^T^	*S. tubercidicus* NBRC 13090^T^	92.25	0.041	44.6
65	*S. violaceusniger* NBRC 13459^T^	*S. sporoclivatus* NBRC 100767^T^	92.09	0.033	43.5
66	*S. violaceusniger* NBRC 13459^T^	*S. melanosporofaciens* DSM 40318^T^	92.08	0.033	43.4
67	*S. coelicoflavus* NBRC 15399^T^	*S. violaceorubidus* NRRL B-16381^T^	92.02	0.021	43.9
68	*S. decoyicus* NRRL 2666^T^	*S. caniferus* NBRC 15389^T^	91.57	0.042	41.6
69	*S. hygroscopicus subsp. hygroscopicus* NBRC 13472^T^	*S. melanosporofaciens* DSM 40318^T^	91.40	0.041	42.8
70	*S. tsukubensis* NRRL 18488^T^	*S. qinzhouensis* SSL-25^T^	91.30	0.035	39.8
71	*S. tirandamycinicus* HNM0039^T^	*S. wuyuanensis* CGMCC 4.7042^T^	91.12	0.033	39.7
72	*S. angustmyceticus* NBRC 3934^T^	*S. decoyicus* NRRL 2666^T^	90.89	0.042	39.4
73	*S. decoyicus* NRRL 2666^T^	*S. libani subsp. libani* NBRC 13452^T^	90.71	0.026	38.7
74	*S. albidochromogenes* DSM 41800^T^	*S. flavidovirens* DSM 40150^T^	90.52	0.050	38.8
75	*S. wuyuanensis* CGMCC 4.7042^T^	*S. spongiicola* HNM0071^T^	90.42	0.040	38.0
76	*S. durhamensis* NRRL B-3309^T^	*S. fodineus* TW1S1^T^	90.33	0.037	38.1
77	*S. platensis* DSM 40041^T^	*S. decoyicus* NRRL 2666^T^	90.20	0.039	37.0
78	*S. hyaluromycini* NBRC 110483^T^	*S. humi* MUSC 119^T^	90.15	0.042	37.3
79	*S. platensis* DSM 40041^T^	*S. caniferus* NBRC 15389^T^	90.08	0.049	36.5
80	*S. decoyicus* NRRL 2666^T^	*S. inhibens* NEAU-D10^T^	90.00	0.050	36.6

**FIGURE 1:**
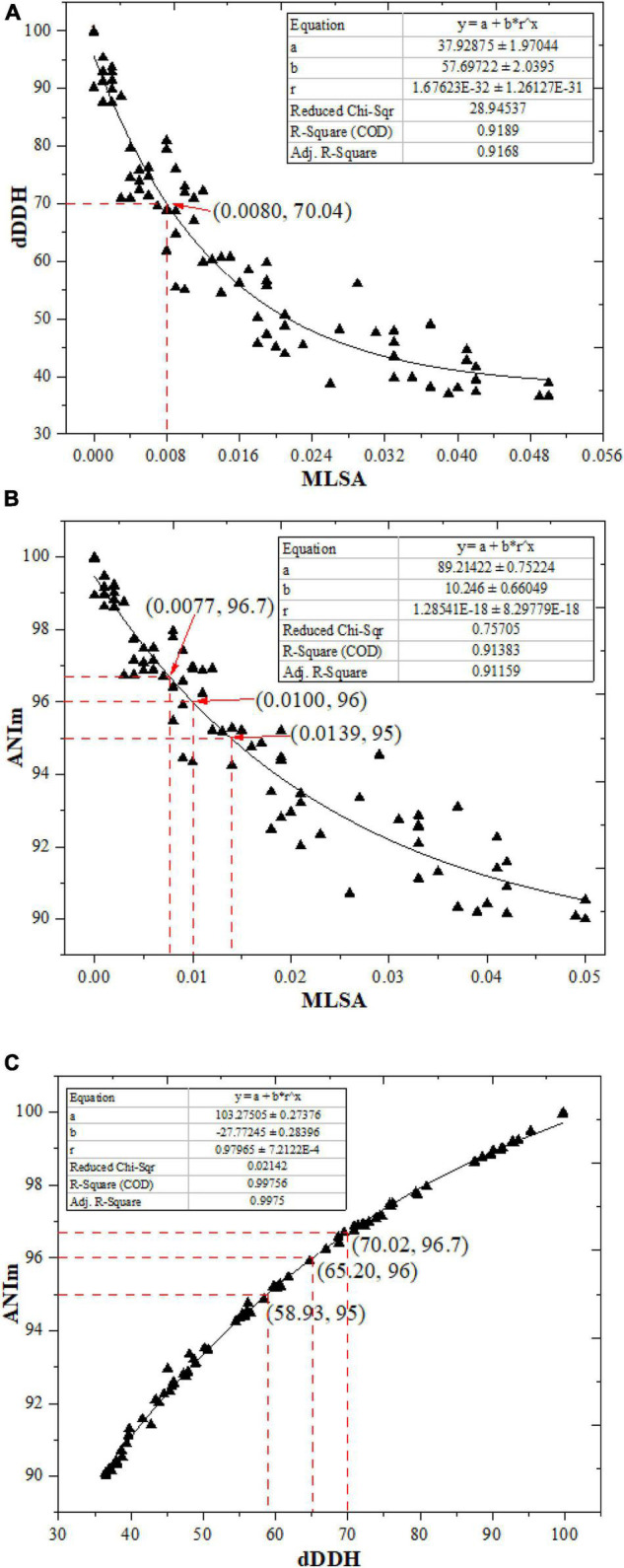
The correlations between ANIm, dDDH, and MLSA from the 80 pairs of *Streptomyces* species (including heterotypic synonyms).

Based on the above analysis, the correlation between dDDH and MLSA, and that between ANIm and MLSA from the aforementioned 80 strain pairs were evaluated by an exponential regression model in order to obtain a more reliable boundary value of MLSA in delineating *Streptomyces* species. As can be seen in Figure 1A, a 70% dDDH value recommended to delineate species approximately corresponded to a MLSA value of 0.008. Theoretically, the MLSA value should decrease with the increase of dDDH value in the light of the putative boundary of 70% dDDH for species circumscriptions. However, in the present work, there were seven scatter points that deviated from this rule. Therefore, the MLSA value of 0.008 could not be simply used as the boundary for *Streptomyces* species circumscriptions. Similarly, it may also be clear from Figure 1B that the proposed 95∼96% ANIm value for delineating species approximately corresponded to a MLSA distance range from 0.010 to 0.014. These results suggested that a certain MLSA value could not be used alone as the threshold for the definition of *Streptomyces* species. Then, what is a more reasonable MLSA value used for defining a *Streptomyces* species? From Table 1, we found that when the MLSA distance value was greater than or equal to 0.014, each strain pair represented the different genomic species; when the MLSA distance value was less than 0.008, the ANIm and dDDH values between each strain pair (except *S. canarius* JCM 4733^T^ and *S. corchorusii* DSM 40340^T^) were more than the 95∼96% and 70% cut-off points recommended for delineating species, respectively. So, each pair should represent the same genomic species except for *S. canarius* and *S. corchorusii* whose taxonomic relationship needed be reevaluated because the dDDH value between them was 69.5%, below 70% boundary point a little, while ANIm value was 96.69%, higher than 95∼96% boundary point. When the MLSA value was between 0.008 and 0.014 (not included), there were seven strain pairs (mentioned above) in which ANIm and dDDH values in each pair were greater than the corresponding thresholds generally accepted by microbial taxonomists, suggesting each pair should represent the same genomic species. In addition, there were nine strain pairs, i.e., *S. albidoflavus* and *S. koyangensis*, *S. bauhiniae* and *S. seoulensis*, *S. rhizosphaericola* and *S. cavourensis*, *S. longwoodensis* and *S. lasalocidi*, *S. longwoodensis* and *S. galbus*, *S. galbus* and *S. lasalocidi*, *S. mirabilis* and *S. olivochromogenes*, *S. chartreusis* and *S. osmaniensis*, and *S. castelarensis* and *S. melanosporofaciens*, whose dDDH values were slightly below the threshold of 70%, suggesting each pair should represent the different genomic species. Nevertheless, there were at least four pairs among the foregoing nine strain pairs, for example, *S. albidoflavus* and *S. koyangensis*, *S. mirabilis* and *S. olivochromogenes*, *S. chartreusis* and *S. osmaniensis*, and *S. castelarensis* and *S. melanosporofaciens*, whose ANIm values were slightly greater than the threshold of 95∼96%, suggesting each pair should belong to the same genomic species. Consequently, what are the reasons for the aforesaid contradictory result? In addition, what is the taxonomic relationship between two strains whose dDDH or ANIm values are near the critical points?

**FIGURE 2:**
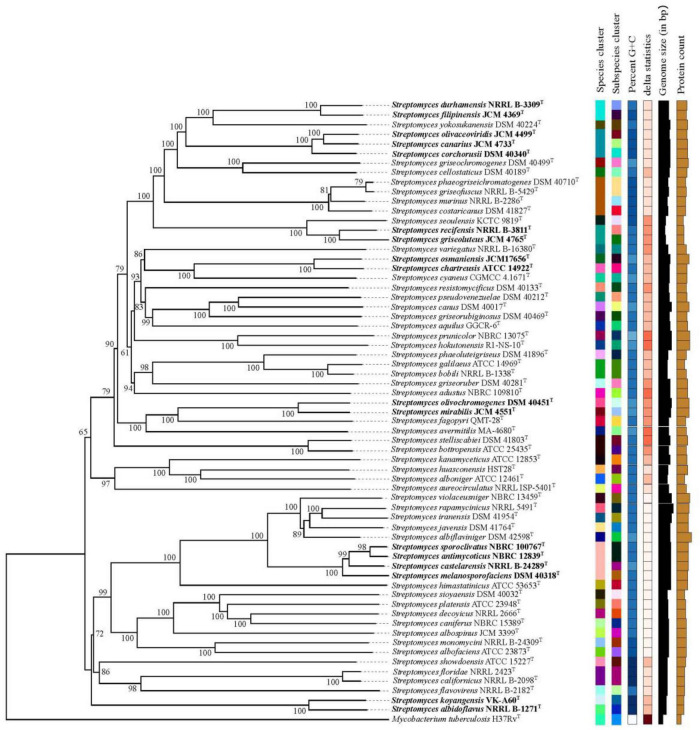
Phylogenomic tree of five pairs of *Streptomyces* species and related reference strains. The numbers above branches are GBDP pseudo-bootstrap support values > 60% from 100 replications, with an average branch support of 96.0%. The tree was rooted at the midpoint (Farris, 1972).

To answer these problems, on the one hand, the correlation between ANIm and dDDH from the aforementioned 80 strain pairs were evaluated by an exponential regression model. It is shown in Figure 1C, the ANIm value revealed an extremely high correlation (*R*^2^ = 0.99756) with the dDDH value, further supporting that ANI can accurately replace DDH values for strains whose genome sequences are available (Goris et al., 2007). However, a 70% dDDH value did not correspond to a 95∼96% ANIm value, but to a ANIm value of approximately 96.7%. Thus, the above contradiction can be well explained based on this corresponding relation. On the other hand, the taxonomic relationships of the five strain pairs (*S. canarius* and *S. corchorusii*, *S. albidoflavus* and *S. koyangensis*, *S. mirabilis* and *S. olivochromogenes*, *S. chartreusis* and *S. osmaniensis*, and *S. castelarensis* and *S. melanosporofaciens*) were reevaluated by using a polyphasic taxonomic approach. At present, it has become a generally accepted principle by biologists to classify living organisms according to the level of phylogenetic correlation since the birth of evolution theory (Ward, 1998). In current prokaryote taxonomy, phylogenetic analysis based on 16S rRNA gene sequences plays a key role in species discrimination. However, there have been evidence that phylogenomic analysis exhibits better resolution than phylogenetic analysis based on 16S rRNA gene sequences (Rodriguez-R et al., 2018; Duchêne, 2021). In the present work, phylogenetic analysis indicated that there were three strain pairs, i.e., *S. albidoflavus* CGMCC 4.1291^T^ and *S. koyangensis* JCM 14915^T^, *S. chartreusis* CGMCC 4.1639^T^ and *S. osmaniensis* JCM 17656^T^, and *S. mirabilis* CGMCC 4.7010^T^ and *S. olivochromogenes* CGMCC 4.2000^T^, in which each pair did not belong to the same species cluster according to the labeled color in the phylogenomic tree (Figure 2), suggesting that these six *Streptomyces* species should represent different genomic species. This result has been further confirmed by differential comparisons of cultural, physio-biochemical and chemotaxonomic characteristics in each pair (Supplementary Tables 2–4); with regard to the remaining two pairs, i.e., *S. canarius* CGMCC 4.1581^T^ and *S. corchorusii* CGMCC 4.1592^T^, and *S. castelarensis* CGMCC 4.3570^T^ and *S. melanosporofaciens* CGMCC 4.1742^T^, strains within each pair should belong to the same genomic species according to the phylogenomic clustering patterns (Figure 2). This result could also be confirmed by the facts shown in Supplementary Tables 5–7, the vast majority of phenotypic features of each strain pair were very similar with only a few exceptions. For example, as far as the former pair was concerned, milk coagulation, milk peptization and α-mannosidase activity were negative for strain CGMCC 4.1581^T^, while positive for strain CGMCC 4.1592^T^; cellular fatty acids such as *iso*-C_19:0_, *anteiso*-C_19:0_ and C_20:0_ were detected for strain CGMCC 4.1592^T^, while not for strain CGMCC 4.1581^T^; the major menaquinone in strain CGMCC 4.1592^T^ was MK-9(H_8_), up to 82.0%, while the major menaquinone in strain CGMCC 4.1581^T^ was MK-9(H_6_), only 53.6%. As far as the latter pair was concerned, activities of α-chymotrypsin, β-galactosidase and valine arylamidase were positive for strain CGMCC 4.3570^T^, while negative for strain CGMCC 4.1742^T^; Assimilation of L-Rhamnose was positive for strain CGMCC 4.3570^T^, while negative for strain CGMCC 4.1742^T^; in cellular fatty acids, the percentage composition of Sum In Feature 8 was up to10.5% for strain CGMCC 4.3570^T^, while only 0.5 for strain CGMCC 4.1742^T^; moreover, in cultural characteristics, color of aerial mycelia on ISP2 and ISP6 was, respectively, dark mouse gray and grayish white for strain CGMCC 4.3570^T^, while both white for strain CGMCC 4.1742^T^. The disagreement for phenotypic characteristics between each strain pair representing the same genomic species was probably due to different ecological niches or minor differences in genotype. All these data supported that 70% dDDH or 96.7% ANIm value could act as the threshold value in delineating *Streptomyces* species. But when dDDH or ANIm value between two closely related *Streptomyces* strains was less than but close to 70 or 96.7% cut-off point, the taxonomic status of a novel strain could only be determined by a combination of phenotypic characteristics, chemotaxonomic characteristics and phylogenomic analysis.

## Conclusion

Based on the above analysis, on the one hand, a 70% dDDH value did not corresponded to a 95∼96% ANIm value but approximately to a 96.7% ANIm value in the genus *Streptomyces*. On the other hand, we proposed that when the MLSA distance value between a novel *Streptomyces* strain and a reference strain was less than 0.008, the novel strain could be considered as a heterotypic synonym of the reference strain; when the MLSA distance value was greater than or equal to 0.014, the novel strain could be regarded as a new *Streptomyces* species; when the MLSA distance value was between 0.008 and 0.014 (not included), ANIm or dDDH value between a new strain and a reference strain must be calculated in order to determine the taxonomic status of a novel strain. Although 70% dDDH or 96.7% ANIm value could act as the threshold value in delineating *Streptomyces* species, if dDDH or ANIm value was less than but close to 70 or 96.7% cut-off point, the taxonomic status of a novel strain could only be determined by a combination of phenotypic characteristics, chemotaxonomic characteristics and phylogenomic analysis.

## Taxonomic Consequences: Emendations

### *Streptomyces melanosporofaciens* Arcamone et al., 1959 (Approved Lists 1980) Is a Later Heterotypic Synonym of *Streptomyces antimycoticus* Waksman, 1957 (Approved Lists 1980)

In the present work, the MLSA distance value between *S. antimycoticus* NBRC 12839^T^ and *S. melanosporofaciens* DSM 40318^T^ is 0.01, higher than the boundary value of 0.008, but the ANIm and dDDH values between them are 96.9% and 72.0, respectively, greater than the 96.7 and 70% cut-off points recommended for delineating species, supporting that they represent the same genomic species. On the basis of these data and rule 42 of the Bacteriological Code (Parker et al., 2019), we propose that *S. melanosporofaciens* is a heterotypic synonym of *S. antimycoticus*.

The description is as given by Komaki and Tamura (2020).

### Emended Description of *Streptomyces filipinensis* (Approved Lists 1980)

Heterotypic synonym: *Streptomyces durhamensis* Gordon and Lapa, 1966 (Approved Lists 1980).

In the present work, the MLSA distance value between *S. filipinensis* JCM 4369^T^ and *S. durhamensis* NRRL B-3309^T^ is 0.01, higher than the boundary value of 0.008, but the ANIm and dDDH values between them are 96.97 and 72.9%, respectively, greater than the 96.7 and 70% cut-off points recommended for delineating species, supporting that they represent the same genomic species. On the basis of these data and rule 42 of the Bacteriological Code, we propose that *S. durhamensis* is a heterotypic synonym of *S. filipinensis*.

The description is as given by Ammann et al. (1955) with the following modification. The G + C content of the type-strain genome is 71.8%, its approximate size 9.03 Mbp, its GenBank deposit SAMD00245426.

### Emended Description of *Streptomyces griseoluteus* (Approved Lists 1980)

Heterotypic synonym: *Streptomyces recifensis* (Gonçalves de Lima et al., 1955) Falcão de Morais et al., 1957 (Approved Lists 1980).

In the present work, the ANIm, dDDH and MLSA distance values between *S. recifensis* NRRL B-3811^T^ and *S. griseoluteus* JCM 4765^T^ are 96.86, 72.4, and 0.005, respectively, far away from the 96.7%, 70%, and 0.008 cut-off points recommended for delineating species, supporting that they represent the same genomic species. On the basis of these data and rule 42 of the Bacteriological Code, we propose that *S. recifensis* is a heterotypic synonym of *S. griseoluteus*.

The description is as given Umezawa et al. (1950) with the following modification. The G + C content of the type-strain genome is 71.6%, its approximate size 6.51 Mbp, its GenBank deposit SAMD00245512.

### Emended Description of *Streptomyces olivaceoviridis* (Preobrazhenskaya and Ryabova, 1957) (Approved Lists 1980)

Heterotypic synonym: *Streptomyces corchorusii* Ahmad and Bhuiyan, 1958 (Approved Lists 1980) and *Streptomyces canarius* Vavra and Dietz, 1965 (Approved Lists 1980).

In the present work, the ANIm, dDDH and MLSA distance values between *S. olivaceoviridis* JCM 4499^T^ and *S. corchorusii* DSM 40340^T^ are 96.88, 71.4 and 0.006, respectively, above the 96.7%, 70% and 0.008 cut-off points recommended for delineating species, supporting that they represent the same genomic species. Meanwhile, the ANIm and dDDH values between *S. canarius* JCM 4733^T^ and *S. corchorusii* DSM 40340^T^ are 96.69 and 69.5%, respectively, near the 96.7 and 70% boundary points, but the MLSA distance value of them is 0.007, below the 0.008 boundary value recommended for delineating species. In addition, this result is further confirmed by the clustering patterns resulting from phylogenomic analysis. Labeda et al. (2017) also recognized that *S. corchorusii* NRRL B-12289^T^ is a later heterotypic synonym of *S. olivaceoviridis* NRRL B-12280^T^. Thus, On the basis of these data and rule 42 of the Bacteriological Code, we propose that *S. corchorusii* and *S. canaries* are latter heterotypic synonyms of *S. olivaceoviridis*.

The description is as given by Pridham et al. (1958) with the following modification. The G + C content of the type-strain genome is 72.1%, its approximate size 9.53 Mbp, its GenBank deposit SAMD00245462.

## Data Availability Statement

The datasets presented in this study can be found in online repositories. The names of the repository/repositories and accession number(s) can be found in the article/Supplementary Material.

## Author Contributions

JG and YZ: revising the manuscript critically for important intellectual content. SH, YW, LF, and YX: acquisition of data. XT: analysis and interpretation of data. All authors contributed to the article and approved the submitted version.

## Conflict of Interest

The authors declare that the research was conducted in the absence of any commercial or financial relationships that could be construed as a potential conflict of interest.

## Publisher’s Note

All claims expressed in this article are solely those of the authors and do not necessarily represent those of their affiliated organizations, or those of the publisher, the editors and the reviewers. Any product that may be evaluated in this article, or claim that may be made by its manufacturer, is not guaranteed or endorsed by the publisher.

## Footnotes

1 www.bacterio.net/genus/Streptomyces and www.ncbi.nlm.nih.gov/assembly/?term=Streptomyces

## Abbreviations

ANI: average nucleotide identity
dDDH: digital DNA–DNA hybridization
MLSA: multi-locus sequence analysis
CGMCC: China General Microbiological Culture Collection Centre
JCM: Japan Collection of Microorganisms
ISP: International *Streptomyces* Project.
