# Supplementary material for: New Insights Into the Threshold Values of Multi-Locus Sequence Analysis, Average Nucleotide Identity and Digital DNA–DNA Hybridization in Delineating Streptomyces Species
Source: Front Microbiol. 2022 May 31;13:910277. doi: 10.3389/fmicb.2022.910277 (PMC9195134; doi:10.3389/fmicb.2022.910277)
Supplement: Supplementary file 1 [file Data_Sheet_1.PDF]

New insights into the threshold values of multi-locus sequence analysis, average nucleotide identity and digital DNA–DNA hybridization in delineating *Streptomyces* species

Siren Hu[1]†, Kaiqin Li[1]†, Yifei Zhang[1], Yinfeng Wang[1], Li Fu[1], Yan Xiao[1], Xinke Tang[1], and Jian Gao[1,2]

[1]School of Life Science, Hunan University of Science and technology, Xiangtan 411201, People's Republic of China;

[2]Key Laboratory of Ecological Remediation and Safe Utilization of Heavy Metal-Polluted Soils, College of Hunan Province, Xiangtan 411201, People's Republic of China.

†These authors contributed equally to this work.

Table S1 The assembly accessions of all type-strain genomes of the genus *Streptomyces* in the current work.

Table S2 Differential comparison of phenotypic characteristics of *S. albidoflavus* CGMCC 4.1291^T and *S. koyangensis* JCM 14915^T

Note: Sum In Feature 3, $C_{16:1}$ ω7c/$C_{16:1}$ ω6c; Sum In Feature 5, $C_{18:2}$ ω6,9c/ante-$C_{18:0;}$ Sum In Feature 8, $C_{18:1}$ ω7c; Sum In Feature 9, iso-$C_{17:1}$ ω9c or $C_{16:0}$ 10-methyl.

Table S3 Differential comparison of phenotypic characteristics of *S. chartreusis* CGMCC 4.1639^T and *S. osmaniensis* JCM 17656^T

Table S4 Differential comparison of phenotypic characteristics of *S. mirabilis* CGMCC 4.7010^T and *S. olivochromogenes* CGMCC 4.2000^T

Note: Sum In Feature 6, $C_{18:2}$ cis9,12/$C_{18:0}$ a.

Table S5 A comparison of phenotypic characteristics of *S. canarius* CGMCC 4.1581^T and *S. corchorusii* CGMCC 4.1592^T

Note: Ara, arabinose; Glc, glucose; Rib, ribose; Xyl, xylose; DPG, diphosphatidylglycerol; PE, phosphatidylethanolamine; PIM, phosphatidylinositol mannosides; PI, phosphatidylinositol; NPG, phospholipids of unknown structure containing glucosamine; PLS, phosphatidylinositol.

Table S6 A comparison of phenotypic characteristics of *S. castelarensis* CGMCC 4.3570^T and *S. melanosporofaciens* CGMCC 4.1742^T

Table S7 A comparison of culture characteristics of *S. chartreusis* CGMCC 4.1639^T, *S. osmaniensis* JCM 17656^T, *S. mirabilis* CGMCC 4.7010^T, *S. olivochromogenes* CGMCC 4.2000^T, *S. albidoflavus* CGMCC 4.1291^T, *S. koyangensis* JCM 14915^T, *S. canarius* CGMCC 4.1581^T, *S. corchorusii* CGMCC 4.1592^T, *S. castelarensis* CGMCC 4.3570^T and *S. melanosporofaciens* CGMCC 4.1742^T

Table S1

Species	DB accession number	Species	DB accession number
S. achromogenes NRRL B-2120^{T}	GCA_000720835.1	*S. rubradiris* JCM 4955^{T}	GCA_014656255.1
S. albidochromogenes DSM 41800^{T}	GCA_005981965.1	*S. albidoflavus* NRRL B-1271^{T}	GCA_000719955.1
S. albus NRRL B-1811^{T}	GCA_000725885.1	*S. almquistii* NRRL B-1685^{T}	GCA_001418125.1
S. angustmyceticus NBRC 3934^{T}	GCA_009176265.1	*S. antibioticus* DSM 40234^{T}	GCA_001514065.1
S. antimycoticus NBRC 12839^{T}	GCA_005405925.1	*S. antioxidans* MUSC 164^{T}	GCA_000968685.2
S. aquilus GGCR-6^{T}	GCA_003955715.1	*S. asterosporus* DSM 41452^{T}	GCA_006716135.1
S. aureofaciens ATCC 10762^{T}	GCA_001188955.3	*S. aureorectus* DSM 41692^{T}	GCA_014138545.1
S. avellaneus NRRL B-3447^{T}	GCA_000721255.1	*S. bauhiniae* Bv016^{T}	GCA_004784475.1
S. bobili NRRL B-1338^{T}	GCA_002154575.1	*S. bottropensis* ATCC 25435^{T}	GCA_000383595.1
S. californicus NRRL B-2098^{T}	GCA_000717645.1	*S. calvus* CECT 3271^{T}	GCA_014138735.1
S. canarius JCM 4733^{T}	GCA_014650735.1	*S. caniferus* NBRC 15389^{T}	GCA_009811555.1
S. castelarensis NRRL B-24289^{T}	GCA_002154275.1	*S. cavourensis* DSM 41795^{T}	GCA_006788935.1
S. chartreusis ATCC 14922^{T}	GCA_008704715.1	*S. coelicoflavus* NBRC 15399^{T}	GCA_003112555.1
S. corchorusii DSM 40340^{T}	GCA_001514055.1	*S. costaricanus* DSM 41827^{T}	GCA_014138855.1
S. diacarni LHW51701^{T}	GCA_003323715.1	*S. ardesiacus* NBRC 15402^{T}	GCA_003112575.1
S. decoyicus NRRL 2666^{T}	GCA_001270575.1	*S. durhamensis* NRRL B-3309^{T}	GCA_000725475.1
S. endus NBRC 12859^{T}	GCA_001553475.1	*S. filipinensis* JCM 4369^{T}	GCA_014649495.1
S. flavidovirens DSM 40150^{T}	GCA_000429085.1	*S. flavovariabilis* NRRL B-16367^{T}	GCA_000725785.1
S. floridae NRRL 2423^{T}	GCA_000717665.1	*S. fodineus* TW1S1^{T}	GCA_001735805.1
S. galbus JCM 4639^{T}	GCA_014650535.1	*S. galilaeus* ATCC 14969^{T}	GCA_008704575.1
S. geysiriensis JCM 4962^{T}	GCA_014651095.1	*S. glebosus* NBRC 13786^{T}	GCA_009811575.1
S. griseofuscus NRRL B-5429^{T}	GCA_000718315.1	*S. griseoluteus* JCM 4765^{T}	GCA_004784465.1
S. griseorubens JCM 4383^{T}	GCA_014649615.1	*S. hawaiiensis* ATCC 12236^{T}	GCA_004803895.1
S. helvaticus DSM 40431^{T}	GCA_005981965.1	*S. humi* MUSC 119^{T}	GCA_001005085.2
S. hyaluromycini NBRC 110483^{T}	GCA_002217755.1	*S. hygroscopicus* subsp. *hygroscopicus* NBRC 13472^{T}	GCA_001553455.1
S. iakyrus NRRL ISP-5482^{T}	GCA_000717055.1	*S. inhibens* NEAU-D10^{T}	GCA_003389455.1
S. koyangensis VK-A60^{T}	GCA_003428925.1	*S. lasalocidi* X-537^{T}	GCA_005280215.1
S. libani subsp. *libani* NBRC 13452^{T}	GCA_009811595.1	*S. longwoodensis* DSM 41677^{T}	GCA_001514125.1
S. matensis JCM 4277^{T}	GCA_014649175.1	*S. mediolani* NRRL WC-3934^{T}	GCA 000721685.1
S. melanosporofaciens DSM 40318^{T}	GCA_900105695.1	*S. mirabilis* JCM 4551^{T}	GCA_014650275.1
S. murinus NRRL B-2286^{T}	GCA_002154555.1	*S. ochraceiscleroticus* NRRL ISP-5594^{T}	GCA_000720485.1
S. olivaceoviridis JCM 4499^{T}	GCA_014650115.1	*S. olivochromogenes* DSM 40451^{T}	GCA_001514115.1
S. osmaniensis JCM17656^{T}	GCA_018596355.1	*S. parvus* NRRL B-1455^{T}	GCA_008632535.1
S. phaeogriseichromatogenes DSM 40710^{T}	GCA_014138905.1	*S. phaeoluteigriseus* DSM 41896^{T}	GCA_001896135.2
S. platensis DSM 40041^{T}	GCA_002119195.1	*S. plicatus* JCM 4504^{T}	GCA_014650135.1
S. puniceus NRRL ISP-5083^{T}	GCA_000718695.1	*S. qaidamensis* S10^{T}	GCA_001611795.1
S. qinzhouensis SSL-25^{T}	GCA_007856155.1	*S. recifensis* NRRL B-3811^{T}	GCA_002154615.1
S. reniochalinae LHW50302^{T}	GCA_003323735.1	*S. rhizosphaericola* 1AS2c^{T}	GCA_004794175.1
S. rubrogriseus NBRC 15455^{T}	GCA_003112595.1	*S. sedi* JCM 16909^{T}	GCA_006335015.1
S. seoulensis KCTC 9819^{T}	GCA_004328625.1	*S. spongiicola* HNM0071^{T}	GCA_003122365.1
S. sporoclivatus NBRC 100767^{T}	GCA_009936315.1	*S. stelliscabiei* DSM 41803^{T}	GCA_014873495.1
S. sulphureus DSM 40104^{T}	GCA_000381025.1	*S. tirandamycinicus* HNM0039^{T}	GCA_003097515.1
S. tsukubensis NRRL 18488^{T}	GCA_003932715.1	*S. tubercidicus* NBRC 13090^{T}	GCA_009811635.1
S. tuirus JCM 4255^{T}	GCA_014701095.1	*S. variegatus* NRRL B-16380^{T}	GCA_000955965.1
S. vinaceusdrappus JCM 4529	GCA_014650215.1	*S. violaceorubidus* NRRL B-16381^{T}	GCA_000717995.1
S. violaceusniger NBRC 13459^{T}	GCA_005405945.1	*S. violens* NRRL ISP-5597^{T}	GCA_000717745.1
S. wuyuanensis CGMCC 4.7042^{T}	GCA_900103455.1	*S. xiaopingdaonensis* DUT 180^{T}	GCA_000262345.1
S. zhaozhouensis CGMCC 4.7095^{T}	GCA_900230195.1		

Table S2

Characteristics	Strain CGMCC 4.1291^{T}	Strain JCM 14915^{T}
Aerial mycelia on ISP2	Drab-Gary	White
Aerial mycelia on ISP4	White	Gary
Aerial mycelia on ISP7	White	Cartridge Buff
Substrate mycelia on ISP7	Olive Lake	Buffy Citrine
H_2S production	–	+
Tween 80	–	+
3%H_2O_2	+	–
Growth at/with:		
pH	6.0-12.0	6.0-14.0
Temperature (℃)	10-30	10-37
NaCl tolerance (%, w/v)	7	10
API ZYM test:		
Acid phosphatase	+	–
Cystine arylamidase	+	–
Esterase lipase (C8)	-	+
N-acetyl-β-glucosaminidase	-	+
Assimilation of sole carbon sources (0.5%, w/v):		
L-Arabinose	–	+
D-Fructose	–	+
D-Mannitol	–	+
Raffinose	+	–
L-Rhamnose	–	+
D-Xylose	–	+
Cellular fatty acids composition (>1.0%):		
iso-$C_{14:0}$	9.5%	5.9%
anteiso-$C_{15:0}$	18.3%	9.0%
iso-$C_{15:0}$	6.8%	5.0%
iso-$C_{16:0}$	27.5%	28.7%
$C_{16:0}$	6.3%	8.8%
iso-$C_{16:1}$ H	4.1%	8.9%
anteiso-$C_{17:0}$	3.5%	3.9%
iso-$C_{17:0}$	1.5%	1.5%
$C_{17:0}$ cyclo	3.1%	5.6%
anteiso-$C_{17:1}$ ω9c	5.4%	5.2%
$C_{18:0}$	(0.7%)	2.8%
iso-$C_{18:1}$ H	(0.7%)	1.8%
$C_{18:1}$ ω9c	1.2%	1.3%
Sum In Feature 3	3.4%	2.6%
Sum In Feature 5	1.4%	(0.2%)
Sum In Feature 8	(0.5%)	1.7%
Sum In Feature 9	1.6%	1.8%

Table S3

Characteristics	Strain CGMCC 4.1639^T	Strain JCM 17656^T
Aerial mycelia on ISP2	Mineral Gray	Pale Green-Blue Gray
Aerial mycelia on ISP3	Puritan Gray	Light Celandine Green
Aerial mycelia on ISP4	Puritan Gray	Light Celandine Green
Substrate mycelia on ISP4	Light Drab	Dark-Green
Soluble pigment on ISP5	None	Brown
Aerial mycelia on ISP7	Grey	White
Soluble pigment on ISP7	None	Brown
Degradation of casein	+	–
Tween 80	–	+
Growth at/with:		
pH	5.0-12.0	6.0-10.0
Temperature (℃)	15-37	15-40
NaCl tolerance (%, w/v)	4	5
API ZYM test:		
Trypsin	+	–
Assimilation of sole carbon sources (0.5%, w/v):		
Cellulose	–	+
glucose	–	+
sorbitol	–	+
D-xylose	–	+
Cellular fatty acids composition (>1.0%):		
iso-$C_{14:0}$	3.4%	7.4%
$C_{14:0}$	(0.3%)	3.2%
anteiso-$C_{15:0}$	8.5%	14.4%
iso-$C_{15:0}$	14.9%	7.1%
$C_{15:0}$	(not detected)	5.5%
iso-$C_{16:0}$	27.1%	23.5%
$C_{16:0}$	5.3%	17.2%
iso-$C_{16:1}$ H	7.1%	1.8%
$C_{16:1}$ cis9	(not detected)	6.8%
anteiso-$C_{17:0}$	5.1%	3.3%
iso-$C_{17:0}$	4.9%	(0.9%)
$C_{17:0}$ cyclo	(0.6%)	1.4%
anteiso-$C_{17:1}$ C	(not detected)	1.1%
anteiso-$C_{17:1}$ ω9c	3.3%	(not detected)
Sum In Feature 3	4.1%	(not detected)
Sum In Feature 5	1.3%	(not detected)
Sum In Feature 9	8.8%	(not detected)

Table S4

Characteristics	Strain CGMCC 4.7010^T	Strain CGMCC 4.2000^T
Aerial mycelia on ISP2	Pallid Neutral Gray	None
Aerial mycelia on ISP3	Light Olive-Gray	None
Aerial mycelia on ISP4	Pallid Neutral Gray	None
Substrate mycelia on ISP4	Buffy Citrine	Primrose Yellow
Aerial mycelia on ISP5	Pallid Neutral Gray	None
Aerial mycelia on ISP7	Olive-Gray	None
Substrate mycelia on ISP7	Dark Grayish Olive	Dresden Brown
Starch hydrolysis	+	–
Gelatin liquefaction	+	–
Hydrolysis of aesculin	–	+
Milk peptization	–	+
3%H_2O_2	–	+
Growth at/with:		
Temperature (℃)	4-37	10-37
NaCl tolerance (%, w/v)	7	5
API ZYM test:		
Esterase(C4)	+	–
esterase lipase (C8)	+	–
β-glucosidase	+	–
Assimilation of sole carbon sources (0.5%, w/v):		
D-Mannitol	–	+
L-rhamnose	–	+
D-trehalose	–	+
D-Xylose	+	–
Cellular fatty acids composition (>1.0%):		
iso-$C_{14:0}$	4.2%	3.7%
$C_{14:0}$	1.1%	(0.8%)
anteiso-$C_{15:0}$	8.3%	23.9%
iso-$C_{15:0}$	2.8%	9.5%
$C_{15:0}$	1.1%	(not detected)
iso-$C_{16:0}$	6.3%	21.8%
$C_{16:0}$	15.7%	6.7%
$C_{16:1}$ cis9	1.1%	(not detected)
iso-$C_{16:1}$ H	(0.2%)	2.8%
anteiso-$C_{17:0}$	(0.9%)	9.5%
iso-$C_{17:0}$	(0.5%)	3.5%
anteiso-$C_{17:1}$ ω9c	(not detected)	3.9%
$C_{18:0}$	(0.8%)	1.0%
$C_{18:1}$ cis9	22.5%	(not detected)
$C_{18:1}$ ω9c	(not detected)	1.0%
Sum In Feature 3	(not detected)	3.1%
Sum In Feature 6	29.9%	(not detected)
Sum In Feature 9	(not detected)	3.6%
Menaquinones:		
	MK-9(1.5%)	
	MK-9(H_2) (2.6%)	MK-9(H_2) (10.4%)
	MK-9(H_4) (15.0%)	MK-9(H_4) (27.5%)
	MK-9(H_6) (40.8%)	MK-9(H_6) (46.5%)
	MK-9(H_8) (37.6%)	MK-9(H_8) (11.9%)

Table S5

Characteristics	Strain CGMCC 4.1581^T	Strain CGMCC 4.1592^T
Spore chain/spore surface	Spiral/Smooth	Spiral/Smooth
Starch hydrolysis	+	+
Gelatin liquefaction	–	–
H_2S production	–	–
Hydrolysis of aesculin	+	+
Nitrate reduction	–	–
Milk coagulation	–	+
Milk peptization	–	+
Tweens (20, 40, 60 and 80)	+	+
3%H_2O_2	+	+
Growth at/with:		
pH	6.0-9.0	6.0-9.0
Temperature (℃)	10-45	10-45
NaCl tolerance (%, w/v)	5	5
API ZYM test:		
Alkaline phosphatase, esterase(C4), esterase lipase (C8)	+	+
N-acetyl-β-glucosaminidase, Naphtol-AS-BI-phosphohydrolase	+	+
α-chymotrypsin, α-fucosidase, lipase (C14)	–	–
α-galactosidase, β-glucosidase, β-glucuronidase, trypsin	–	–
Cystine arylamidase, leucine arylamidase, valine arylamidase	+	+
Acid phosphatase, β-galactosidase, α-glucosidase	+	+
α-mannosidase	–	+
Assimilation of sole carbon sources (0.5%, w/v):		
Glycerol, D-xylose	–	–
L-Arabinose, D-fructose, D-galactose, inositol	+	+
Maltose, D-mannitol, raffinose, L-rhamnose	+	+
Glucose, sucrose, trehalose, sorbitol	+	+
Cellular fatty acids composition (>1.0%):		
iso-$C_{14:0}$	2.0%	2.0%
$C_{14:0}$	1.2%	(0.2%)
anteiso-$C_{15:0}$	27.6%	31.8%
iso-$C_{15:0}$	4.7%	5.1%
$C_{16:0}$	7.2%	3.1%
iso-$C_{16:0}$	11.2%	6.2%
iso-$C_{16:1}$ H	1.2%	1.4%
anteiso-$C_{17:0}$	12.8%	7.8%
iso-$C_{17:0}$	1.7%	4.0%
anteiso-$C_{17:1}$ ω9c	7.5%	2.3%
$C_{18:0}$	1.6%	3.8%
iso-$C_{18:0}$	(0.3%)	1.3%
$C_{18:1}$ ω5c	5.4%	1.6%
$C_{18:1}$ ω9c	4.8%	13.0%
iso-$C_{19:0}$	(not detected)	2.7%
anteiso-$C_{19:0}$	(not detected)	3.0%
$C_{20:0}$	(not detected)	3.0%
Sum In Feature 3	1.9%	(0.7%)
Sum In Feature 5	1.8%	3.4%
Sum In Feature 9	1.6%	(0.8%)
Cell wall amino acids	L,L-DAP, Ala, Asp, Glu, Gly	L,L-DAP, Ala, Asp, Glu,Gly
Whole-cell sugars	Ara, Glc, Rib, Xyl	Ara, Glc, Rib, Xyl
Menaquinones:		
	MK-9(H_2) (3.8%)	MK-9(H_2) (0.4%)
	MK-9(H_4) (7.6%)	MK-9(H_4) (1.0%)
	MK-9(H_6) (53.6%)	MK-9(H_6) (8.7%)
	MK-9(H_8) (30.5%)	MK-9(H_8) (82.0%)
	MK-9(H_{10}) (0.4%)	MK-9(H_{10}) (2.7%)
Major polar lipids	DPG,NPG,PE,PI,PIM,PLS	DPG,NPG,PE,PI,PIM,PLS

Table S6

Characteristics	Strain CGMCC 4.3570^{T}	Strain CGMCC 4.1742^{T}
Spore chain/spore surface	Spiral/Rugose	Spiral/Rugose
Starch hydrolysis	+	+
Gelatin liquefaction	–	–
H_2S production	–	–
Hydrolysis of aesculin	–	–
Nitrate reduction	–	–
Tween 20	–	–
Tweens (40 and 60)	+	+
Tween 80	–	+
3%H_2O_2	+	–
Growth at/with:		
pH	6.0-10.0	6.0-9.0
Temperature (℃)	10-37	10-37
NaCl tolerance (%, w/v)	5	3
API ZYM test:		
Alkaline phosphatase, esterase(C4), naphtol-AS-BI-phosphohydrolase	+	+
Acid phosphatase, N-acetyl-β-glucosaminidase, esterase lipase (C8)	+	+
Cystine arylamidase, lipase (C14), leucine arylamidase	+	+
α-chymotrypsin, β-galactosidase, valine arylamidase	+	–
α-fucosidase, α-mannosidase, trypsin	–	–
α-galactosidase, β-glucuronidase, α-glucosidase, β-glucosidase	–	–
Assimilation of sole carbon sources (0.5%, w/v):		
Sorbitol, sucrose, D-xylose	–	–
L-Arabinose, D-fructose, D-galactose, inositol	+	+
Maltose, D-mannitol, raffinose	+	+
Glucose, glycerol, trehalose	+	+
L-Rhamnose	+	–
Cellular fatty acids composition (>1.0%):		
$C_{12:0}$	1.4%	(0.2%)
iso-$C_{14:0}$	1.9%	2.2%
$C_{14:0}$	(0.7%)	1.4%
anteiso-$C_{15:0}$	10.0%	11.7%
iso-$C_{15:0}$	20.7%	25.6%
iso-$C_{16:0}$	7.5%	10.3%
$C_{16:0}$	11.0%	12.0%
iso-$C_{16:1}$ H	1.0%	(0.9%)
anteiso-$C_{17:0}$	3.2%	4.9%
iso-$C_{17:0}$	6.5%	9.0%
$C_{17:0}$ cyclo	1.0%	(0.9%)
anteiso-$C_{17:1}$ ω9c	1.2%	1.2%
Sum In Feature 3	9.1%	5.4%
Sum In Feature 5	(0.5%)	1.5%
Sum In Feature 8	10.5%	(0.5%)
Sum In Feature 9	6.2%	6.1%
Cell wall amino acids:		
	L,L-DAP, *meso*-DAP, Ala	L,L-DAP, *meso*-DAP, Ala
	Glu, Gly	Glu, Gly
Whole-cell sugars	Glc, Rib	Glc, Rib
Menaquinones:		
	MK-9(H_4) (5.1%)	MK-9(H_4) (12.0%)
	MK-9(H_6) (20.5%)	MK-9(H_6) (26.1%)
	MK-9(H_8) (57.0%)	MK-9(H_8) (50.0%)
	MK-9(H_{10}) (13.4%)	MK-9(H_{10}) (7.7%)
Major polar lipids	DPG,NPG,PE,PI,PIM,PME	DPG,NPG,PE,PI,PIM,PME

Table S7

Characteristics	CGMCC 4.1639^{T}	JCM 17656^{T}	CGMCC 4.7010^{T}	CGMCC 4.2000^{T}	CGMCC 4.1291^{T}	JCM 14915^{T}	CGMCC 4.1581^{T}	CGMCC 4.1592^{T}	CGMCC 4.3570^{T}	CGMCC 4.1742^{T}
AM on ISP2	Mineral Gray	Pale Green-Blue Gray	Pallid Neutral Gray	None	Drab-Gary	White	White	White	Dark Mouse Gray	White
SM on ISP2	Buffy-Citrine	Buffy-Citrine	Grayish Olive	Light Grayish Olive	Burnt Sienna	Burnt Sienna	Aniline Yellow	Aniline Yellow	Cinnamon	Cinnamon
SP on ISP2	None	None	None	None	None	None	None	None	None	None
AM on ISP3	Puritan Gray	Light Celandine Green	Light Olive-Gray	None	Pale Drab-Gary	Pale Drab-Gary	Pallid Quaker Drab	Pallid Quaker Drab	Deep Olive-Gray	Deep Olive-Gray
SM on ISP3	Pale Fluorite Green	Pale Fluorite Green	Deep Grayish Olive	Light Grayish Olive	Light Grayish Olive	Light Grayish Olive	Citron Yellow	Citron Yellow	Massicot Yellow	Massicot Yellow
SP on ISP3	None	None	None	None	None	None	None	None	None	None
AM on ISP4	Puritan Gray	Light Celandine Green	Pallid Neutral Gray	None	White	Gary	Light Olive-Gray	Olive-Gray	Dark Mouse Gray	Pale Gull Gray
SM on ISP4	Light Drab	Dark-Green	Buffy Citrine	Primrose Yellow	Ecru-Olive	Ecru-Olive	Honey Yellow	Honey Yellow	Honey Yellow	Massicot Yellow
SP on ISP4	None	None	None	None	None	None	None	None	None	None
AM on ISP5	Grey	Grey	Pallid Neutral Gray	None	White	White	Pale Gull Gray	Light Gull Gray	Pale Gull Gray	Pale Gull Gray
SM on ISP5	Warm Buff	Warm Buff	Pale Chalcedony Yellow	Pale Chalcedony Yellow	Cream-Buff	Cream-Buff	Light Orange-Yellow	Light Orange-Yellow	Massicot Yellow	Massicot Yellow
SP on ISP5	None	Brown	None	None	None	None	None	None	None	None
AM on ISP6	None	None	None	None	White	White	None	None	White	White
SM on ISP6	Ivory Yellow	Ivory Yellow	Deep Olive-Buff	Deep Olive-Buff	Russet	Russet	Yellow Ocher	Yellow Ocher	Antimony Yellow	Antimony Yellow
SP on ISP6	Brown	Brown	None	None	Kaiser Brown	Kaiser Brown	None	None	None	None
AM on ISP7	Grey	White	Olive-Gray	None	White	Cartridge Buff	Pale Gull Gray	Pallid Neutral Gray	Deep Olive-Gray	Light Olive-Gray
SM on ISP7	Warm Buff	Warm Buff	Dark Grayish Olive	Dresden Brown	Olive Lake	Buffy Citrine	Wax Yellow	Old Gold	Honey Yellow	Natal Brown
SP on ISP7	None	Brown	None	None	None	None	None	None	Wood Brown	Wood Brown

Note: AM, aerial mycelia; SM, substrate mycelia; SP, Soluble pigment.
